# Predictors of 25-hydroxyvitamin D status among individuals with metabolic syndrome: a cross-sectional study

**DOI:** 10.1186/s13098-018-0346-1

**Published:** 2018-06-04

**Authors:** Séphora Louyse Silva Aquino, Aline Tuane Oliveira da Cunha, Hermilla Torres Pereira, Erika Paula Silva Freitas, Ana Paula Trussardi Fayh, Josivan Gomes Lima, Severina Carla Vieira Cunha Lima, Karine Cavalcanti Maurício Sena-Evangelista, Lucia Fátima Campos Pedrosa

**Affiliations:** 1 Postgraduate Nutrition Program, Center for Health Sciences, Federal University of Rio Grande do Norte, Natal, RN 59078-970 Brazil; 2 Postgraduate Program in Health Sciences, Center for Health Sciences, Federal University of Rio Grande do Norte, Natal, RN 59012-570 Brazil; 3 Department of Nutrition, Center for Health Sciences, Federal University of Rio Grande do Norte, Natal, RN 59078-970 Brazil; 4 Department of Clinical Medicine, Endocrine Unit, Federal University of Rio Grande do Norte, Natal, RN 59010-180 Brazil

**Keywords:** Metabolic syndrome, Vitamin D, Sun exposure, Seasonal variation

## Abstract

**Background:**

The risk of metabolic syndrome can be influenced by inadequate vitamin D levels, and exposure to sunlight is the main external source of vitamin D. The present study assessed the influence of environmental, biological, and nutritional factors in relation to seasonal 25-hydroxyvitamin D (25OHD) concentration in individuals with metabolic syndrome.

**Methods:**

This cross-sectional study enrolled 180 individuals with metabolic syndrome aged between 18 and 80 years. The 25OHD concentration was considered the dependent variable; independent variables included age, sex, skin color, use of sunscreen, skin type, sun exposure score, ultraviolet radiation index, geographic location, season, body mass index, waist:hip ratio, waist circumference, parathyroid hormone level, total serum calcium level, and calcium and vitamin D intake.

**Results:**

The average vitamin D in individuals evaluated in summer 32 ± 10 ng/mL was greater than in the winter 26 ± 8 ng/mL (p < 0.017). HDL-cholesterol was the only component of the MetS that differed significantly between the seasons (p < 0.001), showing higher concentrations in autumn 45 ± 8 mg/dL than in summer 35 ± 8 mg/dL. In the multiple regression model, gender, WHR, sun exposure score, and winter vs. summer explained 10% of the variation in 25OHD concentration (p = 0.004).

**Conclusions:**

Sex, waist:hip ratio, sun exposure, and summer season were predictors of 25OHD status among individuals with metabolic syndrome. HDL-cholesterol was the only component of metabolic syndrome that differed significantly between the seasons.

**Electronic supplementary material:**

The online version of this article (10.1186/s13098-018-0346-1) contains supplementary material, which is available to authorized users.

## Background

Metabolic syndrome (MetS) is characterized by the presence of three or more cardiovascular risk factors, and is usually related to the central deposition of fat as well as insulin resistance [1]. Worldwide, it is estimated that 30–40% of the population 65 years of age and older has MetS, due to excessive weight during adulthood [2]. In Brazil, the prevalence rate of MetS has been reported at around 29.6% in individuals aged 19–64 years [3].

Vitamin D deficiency can play a role in the physiopathology of risk factors for MetS and the components thereof, including hypertension, atherogenic dyslipidemia, diabetes mellitus type 2, and central obesity [4, 5]. A systematic review and meta-analysis of 99,745 participants reported statistically significant associations between decrease 25-hydroxyvitamin D (25OHD) levels in adult and elderly individuals, and an increase in cardiovascular diseases, diabetes mellitus type 2 and MetS [6].

Several predictors are involved in changes in vitamin D status in chronic diseases, with seasonality being currently discussed as an important factor, because it has an impact on the behavior and lifestyle of individuals [7]. For example, the winter season has a higher prevalence of MetS, and changes in the components thereof [7, 8]. Seasonal variation in fasting blood glucose and blood pressure was observed in Japanese individuals [9]. The effects of climatic factors on plasma lipid levels have also been reported in the literature [10].

In addition to sun exposure, the serum concentration of 25OHD is also influenced by nutritional status, physical activity, diet, and skin pigmentation [11, 12]. Inadequate 25OHD was observed in overweight individuals, regardless of the degree of obesity [13]. Diet, especially one that is lacking in vitamins and minerals, is considered another risk factor for MetS [14].

Besides natural sources of vitamin D, fortified foods, nutritional supplements and medications, and sun exposure are the main external sources of vitamin D [15]. Ultraviolet-B (UVB) rays (wavelength 290–315 nm) from the sun are absorbed by 7-dehydrocholesterol (7-DHC) present inside epidermal cell plasma membranes, resulting in the production of the intracellular cis, cis pre-vitamin D_3_, which is non-enzymatically isomerized, resulting in vitamin D_3_ [16]. In this sense, compared to lighter skin, darker skin appears to be less efficient in the production of pre-vitamin D_3_, and subsequently, vitamin D_3_, due to the competition between melanin and the 7-DHC for photons of ultraviolet radiation [17, 18].

Therefore, taking into account the known predictors of MetS, and the impact of seasonality thereof, this study aimed to assess the influence of the environmental, biological, and nutritional factors in the seasonal changes of 25OHD among individuals with MetS.

## Methods

### Study design

A cross-sectional study was developed with adult and elderly individuals of both sexes, 18–80 years of age, diagnosed with MetS, and treated at the Endocrinology Clinic of Onofre Lopes University Hospital (HUOL) of the Federal University of Rio Grande do Norte (UFRN), Natal, Brazil. The study was approved by the Research Ethics Committee of HUOL (CAAE n. 13699913.7.0000.5292) and the participants signed informed consent forms indicating their agreement to participate in the research. Individuals with diabetes mellitus type 1 or type 2 who used insulin were excluded, as well as those with altered renal or hepatic function (glomerular filtration rate estimated by modification of diet in renal disease < 60 mL/min; hepatic transaminase levels higher than three times the reference values), decompensated heart failure, those who were pregnant or lactating, those who had used glucocorticoids in the past 3 months, those who were taking anti-epileptics or rifampin, and those had taken calcium or vitamin D supplementation in the past 30 days.

MetS was diagnosed according to criteria from the National Cholesterol Education Program-Adult Treatment Panel III (NCEP-ATP III), which includes the presence of at least three of the following: waist circumference > 102 cm in men and > 88 cm in women; triglyceride levels ≥ 150 mg/dL; high-density lipoprotein (HDL) cholesterol level < 40 mg/dL in men and < 50 mg/dL in women; blood pressure ≥ 130 mmHg or ≥ 85 mmHg, and fasting blood glucose ≥ 100 mg/dL [19].

From August 2013 to December 2015, 3068 patient medical records were screened; of these, 1199 were excluded for not meeting the inclusion criteria, and 1586 patients were not included because they were not diagnosed with MetS. Of the 283 individuals who met the study inclusion criteria, 77 refused to participate in the study or were absent from the endocrinologist appointment; and 26 individuals were lost due to absence of blood collection. Therefore, data collection was complete for 180 participants.

### Anthropometric assessment

The anthropometric evaluation was performed using body mass index (BMI), waist circumference (WC), and waist:hip ratio (WHR). The WC was measured as half the distance between the iliac crest and the lower costal margin and classified according to the NCEP-ATP III criteria [19]. The WHR was calculated using > 0.90 and > 0.85 as the cut-off values for men and women, respectively [20].

### Blood pressure

Systolic and diastolic blood pressure values were measured during the clinical consultation, according to the Brazilian Guidelines of Blood Pressure VI [21].

### Food intake assessment

Data regarding food intake were obtained using the 24 h dietary recall method (R24h), applied twice, between 30 and 45 days. Diet analysis was performed using Virtual Nutri Plus^®^ 2.0 (Keeple Company São Paulo, Brazil) The composition of the culinary preparations was noted in the dietary records for later data capture. The average nutrient intakes were calculated as the mean of the intake values obtained from both R24h. The results of the dietary intake of vitamin D and calcium were expressed as IU/day and mg/day, respectively.

### Sun exposure

Sun exposure was evaluated based on a weekly score equivalent to the individual sun exposure for the previous 7 days. First, the daily score was obtained by measuring the amount of time that each individual spent outdoors versus the amount of exposed skin. The weekly scores were measured, and the resulting sum of these daily scores was defined as the sun exposure. The use of sunscreen in the last 7 days was also assessed. The scores ranged from zero (no exposure) to 56 (maximum exposure) [22].

### Skin pigmentation

Skin color was obtained by self-classification among the five categories adopted by the Brazilian Institute of Geography and Statistics (IBGE): black, mixed (“pardo” in official Portuguese), white, yellow (Asian), and indigenous (Native American) [23]. The skin types were classified according to Fitzpatrick (1988), who classified human skin color into six categories, ranging from type I (fair) to type VI (black) [24].

### Ultraviolet index and seasons

Data from the monitoring and recording of ultraviolet index (UVI) in the city of Natal, RN, Brazil was obtained from the daily publications from the Laboratory of Tropical Environmental Variables (LAVAT), of the National Institute of Spatial Researches, Regional Center of the Northeast (INPE/CRN). The UVI was classified according to World Health Organization (WHO) guidelines [25] into five categories according to UVI intensity: low, < 2; moderate, 3–5; high, 6–7; very high, 8–10, and extreme, ≥ 11.

The participants were distributed in the spring, summer, autumn, and winter seasons based on the half-life of 25OHD corresponding to the 30 days preceding the date of the biochemical examination [26]. Thus, individual participants were categorized into the respective seasons based on the month into which this time interval fell.

### Biochemical tests

Blood samples were collected from the participants after an overnight fast (10–12 h) by standard venipuncture. Fasting blood glucose, HDL-cholesterol, triglyceride, and total calcium levels were measured by colorimetric tests using the test kit from the Wiener lab^®^ (Wiener lab group, Argentina) and via the automated CMD800iX1 (Diamond Diagnostics, Holliston, MA, USA). Low-density lipoprotein (LDL)-cholesterol levels were measured as previously described [27].

The serum concentrations of the parathyroid (PTH) and insulin hormones were determined by chemiluminescent tests using a commercial analysis from Beckman Coulter^®^ (Unicel^®^ DxI800 immunoassay system, CA, USA). Serum concentrations of 25OHD were measured using the chemiluminescent Liaison^®^ test from kit DiaSorin^®^ (Saluggia, Italy).

Lipid profiles were classified according to the NCEP-ATPIII criteria [19]. The established criterion for fasting blood glucose levels was ≥ 100 mg/dL [1]. The reference range for total serum calcium was 8.8–11 mg/dL, and the reference interval for PTH levels was 11–67 pg/mL. The 25OHD levels were considered deficient, insufficient, and sufficient for concentrations ≤ 20, ≥ 21 and ≤ 29, and ≥ 30 ng/mL, respectively [28].

### Statistical analysis

For the descriptive analysis of continuous variables, mean ± standard deviation (SD), means (confidence interval [CI]), or medians (interquartile interval) were calculated as appropriate. The absolute and relative frequencies were calculated for binary and categorical variables.

There were missing data for PTH levels, total serum calcium, use of sunscreen, and skin color. Therefore, methods of multiple imputation data were applied, which follow three main steps: (a) imputation of missing data to obtain complete databases; (b) estimation based on incomplete databases, and (c) a combination of methods. Data were assumed to be missing at random [29].

The 25OHD concentration variation throughout the seasons was examined by one-way analysis of variance (ANOVA). When the results were statistically significant, posthoc tests were performed to assess the differences among seasons. T-tests were used for independent samples, followed by the correction of Bonferroni. For the variables with imputed observations, the analysis between the seasons was performed using F tests (analog of ANOVA) of the univariate linear regression model. When the F test was significant, the P values of the regression coefficients were also corrected by the Bonferroni method.

The potential predictors for the magnitude of the 25OHD concentration were investigated using univariate linear regression models and as well as multiple regression (two or more predictor variables included in the model).

In the univariate linear regression, 15 models were created using 25OHD concentration as a dependent variable and the other variables as predictors. In the multiple regression analysis, seven models were created using 25OHD levels as the dependent variable and the other variables as predictors, as follows: (1) gender, age, use of sunscreen, sun exposure score, season, and WHR; (2) gender, age, use of sunscreen, sun exposure score, season, and BMI; (3) gender, age, use of sunscreen, sun exposure score, season, and WC; (4) age, gender and region; (5) season, UVI, sun exposure score, skin type, skin color, and use of sunscreen; (6) total serum calcium and PTH and (7) vitamin D and calcium intakes (Additional file 1).

The correlation between two variables was assessed by determining Pearson correlation coefficients (r). For variables with imputed observations, the Pearson coefficient was computed using the coefficient of simple linear regression, considering the variables centered in their averages and standardized by the sample standard deviation. Fisher’s exact test and its extensions were used to test the differences between proportions. The statistical significance assumed for all analysis was 5% (two-tailed, p < 0.05). All analyses were performed using Stata 14.0 (Stata Corporation, College Station, TX, USA).

## Results

### Characteristics of the participants

Of the 180 total participants, 141 (78%) were female and 39 (22%) were male, with an average age of 50 ± 12 years. There was no statistically significant variation in the number of MetS components between the seasons (Table 1). The most frequent components of MetS in the participants were elevated WC (88%), elevated blood pressure (79%), low HDL-cholesterol (78%), elevated fasting blood glucose (75%), and high triglyceride levels (54%). The HDL-cholesterol was the only component with significant variation among seasons, showing higher concentrations in the autumn 45 ± 8 mg/dL and lower in the summer 35 ± 8 mg/dL (Table 2).

**Table 1:** Demographic, biological, and environmental characteristics of the individuals according to season

Variables	Seasons				Total (n = 180)	*p* ^c^
	Winter (n = 84)	Spring (n = 50)	Summer (n = 28)	Autumn (n = 18)		
Gender^a^						0.87
Female	66 (79)	39 (78)	23 (82)	13 (72)	141 (78)	
Male	18 (22)	11 (22)	5 (18)	5 (28)	39 (22)	
Age (years)^b^	51 ± 13	49 ± 10	47 ± 13	54 ± 14	50 ± 12	0.22
Number of MetS components^a^						0.33^d^
3 components	43 (51)	21 (42)	10 (36)	6 (33)	80 (44)	
4 components	29 (35)	16 (32)	13 (46)	6 (33)	64 (36)	
5 components	12 (14)	13 (26)	5 (18)	6 (33)	36 (20)	
Self-referred skin color^a^						0.59
Black	6 (7)	4 (8)	–	–	10 (6)	
Mixed	51 (61)	26 (52)	17 (61)	15 (83)	109 (61)	
White	22 (26)	18 (36)	10 (36)	2 (11)	52 (29)	
Yellow	3 (5)	2 (4)	1 (4)	1 (6)	7 (3)	
Indigenous	1 (1)	–	–	–	1 (1)	
Sunscreen^a^						0.25
Do not use	51 (61)	37 (74)	17 (61)	14 (78)	120 (67)	
Always use	33 (39)	12 (24)	11 (39)	4 (22)	60 (33)	
Skin type^a,e^						0.29
I	7 (8)	5 (10)	2 (7)	–	14 (8)	
II	30 (36)	13 (26)	10 (36)	4 (22)	57 (32)	
III	20 (24)	12 (24)	6 (21)	4 (22)	42 (23)	
IV	17 (20)	15 (30)	5 (18)	3 (17)	40 (22)	
V	10 (12)	5 (10)	5 (18)	7 (39)	27 (15)	
UVI^b,f^	5.5 ± 1.4	7.9 ± 2.2	6.8 ± 0.4	5.8 ± 0.4	6.4 ± 1.8	< 0.001

^a^ Data presented as n(%)

^b^Data presented as average ± standard deviation

^c^p, differences between seasons. T-tests were used for independent samples, followed by the correction of Bonferroni and Fisher’s exact test and its extensions were used to test the differences between proportions

^d^Chi square test was used to compare the proportions of the number of components between stations

^e^Skin type according to Fitzpratrick^21^

^f^UVI, Ultraviolet radiation index of the 30 days previous to the biochemical exam

**Table 2:** Anthropometric nutritional status, clinics, and dietary characteristics of the individuals according to the season

Variables	Seasons				Total (*n* = 180)	*p* ^c^
	Winter (n = 84)	Spring (n = 50)	Summer (n = 28)	Autumn (n = 18)		
BMI (kg/m^2^)^a^	32 ± 7	34 ± 6	35 ± 7	34 ± 8	33 ± 7	0.36
WHR^a^	0.97 ± 0.1	0.97 ± 0.1	0.97 ± 0.1	0.97 ± 0.1	0.97 ± 0.1	0.91
WC (cm)^a^	104 ± 14	107 ± 11	108 ± 14	106 ± 14	106 ± 13	0.39
Triglycerides (mg/dL)^b^	164 (126–217)	172 (116–235)	148 (120–188)	163 (135–218)	162 (120–221)	0.81
HDL-cholesterol (mg/dL)^a^	42 ± 9	37 ± 9	35 ± 8	45 ± 8	40 ± 9	< 0.001^d^
Fasting blood glucose (mg/dL)^b^	108 (94–130)	110 (96–125)	106 (95–119)	117 (105–140)	108 (96–129)	0.35
Systolic blood pressure (mm/Hg)^b^	130 (120–140)	132 (122–140)	128 (120–140)	130 (122–140)	130 (120–140)	0.80
Diastolic blood pressure (mm/Hg)^b^	84 (80–90)	89 (83–95)	87 (80–90)	90 (83–93)	87 (80–90)	0.15
25OHD (ng/mL)^a^	26 ± 8	29 ± 10	32 ± 10	30 ± 9	28 ± 9	0.014^e^
Total serum calcium (mg/dL)^a^	10.1 ± 0.7	9.9 ± 0.7	9.4 ± 0.5	9.8 ± 0.4	9.9 ± 0.7	< 0.001^f^
PTH (pg/mL)^b^	35 (25–51)	35 (22–48)	25 (17–34)	36 (20–41)	34 (22–47)	0.06
Vitamin D intake (IU/day)^b^	109 (63–150)	82 (45–141)	80 (60–124)	91 (67–158)	90 (59–146)	0.28
Calcium intake (mg/day)^b^	473 (315–632)	426 (254–560)	409 (245–639)	404 (297–524)	441 (294–593)	0.30

*BMI* body mass index, *WHR* waist:hip ratio, *WC* waist circumference; *PTH* parathyroid hormone

^a^Data presented as average ± standard deviation)

^b^Data presented as median (interquartile interval)

^c^p, difference among seasons. ANOVA was used to compare the variables throughout the seasons. For the variables with imputed observations, the analysis between seasons was performed using F tests (analog of ANOVA)

^d^p, for multiple comparisons: winter vs. spring (p = 0.007), winter vs. summer (p = 0.003), spring vs. autumn (p = 0.012), and summer vs. autumn (p = 0.004)

^e^p, for multiple comparisons: winter vs. spring (p = 0.378), winter vs. summer (p = 0.017), winter vs. autumn (p = 0.581), spring vs. summer (p > 0.99), spring vs autumn (p > 0.99), and summer vs. autumn (p > 0.99)

^f^p, for multiple comparisons: winter vs. spring (p = 0.67), winter vs. summer (p = 0.001), winter vs. autumn (p = 0.16), spring vs. summer (p = 0.012), spring vs. autumn (p > 0.99), and summer vs. autumn (p = 0.73)

Most of the individuals had mixed skin (67%), group II skin type (32%), and did not use sunscreen (66%). The annual UVI average of 6.4 ± 1.8 was high. The UVI differed significantly among seasons, especially between winter and spring (p < 0.001), winter and summer (p = 0.001), spring and summer (p = 0.022), and spring and autumn (p < 0.001), with index variations ranging from moderate to very high (Table 1).

The average BMI was 33 ± 7 kg/m^2^; 77 and 23% had obesity and overweight, respectively. The average overall WC was 106 ± 13 cm, indicating an increased risk for the development of diseases associated with obesity. BMI, WHR, and WC did not show significant statistical differences among the seasons (Table 2).

### Seasonal variations of the 25OHD

The percentage of individuals with 25OHD deficiency and insufficiency was higher in the winter (72%) and lower in the summer (50%). The average 25OHD concentration was 5.59 ng/mL higher in the summer than in the winter (95% CI 1.81–9.38 ng/mL; p = 0.024).

### Predictors of 25OHD concentration

In the simple linear regression model, a statistically significant association with summer (p = 0.003) was observed, explaining 4% of the variability in 25OHD status. Sun exposure score was significantly associated with 25OHD status (p = 0.008). There was no statistically significant association between 25OHD concentration and the co-variables of age, sex, geographic location, skin color, skin type and use of sunscreen, UVI, BMI, WHR, WC, total serum calcium, PTH, and dietary calcium, and vitamin D.

In the multiple regression model, sex, WHR, sun exposure score, and season significantly influenced 25OHD status explaining 10% of the variation in 25OHD status (p = 0.001; Table 3).

**Table 3:** Multiple regression model for the prediction of 25OHD concentrations (ng/mL) in individuals with MetS (n = 180)

25OHD (ng/mL)					
Predictors	Β	SE	95% CI	*p* ^a^	*R* ^2^
Sun exposure score	0.164	0.06	0.05–0.28	0.006	0.104
Seasons					
Winter (Ref.)					
Spring	3.178	1.57	0.08–6.27	0.045	
Summer	5.593	1.92	1.81–9.38	0.004	
Autumn	3.108	2.29	− 1.42–7.64	0.18	
Sex	3.716	1.67	0.43–7.00	0.027	
WHR	− 25.080	11.23	− 47.26–2.89	0.027	
Age (years)	0.0001	0.56	− 0.11–0.11	0.10	
Use of sunscreen					
Do not use (Ref.)					
Always use	2.671	1.41	− 0.11–5.45	0.06	
Sometimes use	− 3.91	8.91	− 21.52–13.69	0.66	

*β* regression coefficient, *SE* standard error, *CI* confidence interval, *Ref.* reference group

^a^p, for multiple comparisons: winter vs. spring, winter vs. summer, winter vs. autumn, spring vs. summer, spring vs. autumn, and summer vs. autumn

Men with MetS had 25OHD concentration 3.71 ng/mL higher than those of women in this study. A 1.0 increment in sun exposure score was associated with a 0.16 ng/mL increase in 25OHD concentration, and the season was an independent predictor of 25OHD status.

## Discussion

In the present study, sex, WHR, sun exposure score, and season were predictors of 25OHD status among individuals with MetS. We found *significantly higher* serum *25OHD* concentrations in the summer compared to the winter. Summertime improvement of vitamin D status was accompanied by certain improved cardiometabolic risk factors, notably serum triglycerides, total cholesterol and BMI, in Iranian children [30]. We speculated that the higher prevalence of inadequate 25OHD levels in the study population might be associated with MetS clinical conditions.

The discovery of the inverse relationship between WHR and 25OHD status suggested that a high fat concentration in the abdominal region may interfere with vitamin D metabolism. This is explained by the liposoluble nature of vitamin D, thus higher fat concentrations in the abdominal region favor the uptake of vitamin D to the adipose tissue, resulting in inadequate 25OHD concentrations. Miñambres et al. found inadequate 25OHD status regardless of the level of obesity, a finding that supports the existence of this inverse correlation between higher abdominal adipose tissue concentration and inadequate 25OHD [13].

Few studies have assessed the influence of the seasons on 25OHD status among individuals living in cities in the northeastern regions of Brazil. Studies conducted on adults and elderly in the city of São Paulo, in the southeastern region of Brazil, reported results similar to those of our study [31, 32]. The positive association between the sun exposure score and 25OHD status can be explained by the presence of 7-DHC in the plasma membrane of epidermal cells; it is a photosensitive molecule that absorbs ultraviolet radiation with a wavelength from 290–315 nm. Following this absorption, the entire metabolic pathway is initiated to activate vitamin D synthesis [8]. This discovery reinforces the utility of the assessment tool used in the study, which offered good accuracy to assess sun exposure.

Sy et al. reported that an increase of 25OHD concentration by approximately 10 ng/mL decreases the risk of developing MetS by 13%. An important point for further discussion is therefore the ideal 25OHD concentration for individuals with MetS [33].

Because the Rio Grande do Norte state is a Brazilian territory with high year-round solar radiation, 25OHD levels were expected to be sufficient in this population, regardless of the season [16]. In the period assessed in the current study, the average environmental UVI ranged from moderate to high in every season, with significant differences between the moderate and high UVI in the winter and summer, respectively. However, 63% of the individuals assessed in this study had inadequate 25OHD levels, the proportion of which increased in the winter. This finding emphasizes the essential roles of the seasons, and sun exposure on the variability in 25OHD status.

More recently, there has been a growing appreciation for the beneficial impact that sunlight has on the cardiovascular system, independent of vitamin D production. Vitamin D could in these circumstances act as a marker for sunlight exposure and its postulated beneficial effects [34].

The seasonality of vitamin D status has been noted in other studies, in addition to reports on the incidence of MetS and its related components [35]. In the present study, seasonal variation was observed in the concentration of HDL-cholesterol, especially in the summer, where the lowest concentrations of this component were noted. A study of 1202 male Japanese subjects reported a higher prevalence of MetS in the winter compared to that of the summer, with similar results for the MetS components such as HDL-cholesterol, systolic and diastolic blood pressure, and fasting blood glucose levels [7]. Summer season was also positively associated with low HDL-C, and MetS in Chinese adults when summer–winter differences in components of MetS were investigated [36].

We can attribute higher concentrations of 25OHD in the summer, along with lower concentrations of HDL-cholesterol, to the fact that 64% of individuals were diagnosed as having more than three of the components of MetS, which represents greater metabolic impairment. Changes in the lipid profile between the seasons can be explained by a set of seasonal changes, such as blood hemodilution during the summer, and blood hemoconcentration in winter, as well as changes in eating habits, and physical activity [37]. The association between low 25OHD status, and low HDL-cholesterol concentrations has been discussed in some studies but are still controversial. Data from the Tromsø Study showed in adults a strong and positive association between serum 25(OH)D and HDL. However, the cause of this association still remains unknown [38, 39]. Our study did not focus on evaluating the association among vitamin D and lipid profile or the other components of the MetS.

The higher 25OHD levels in the male participants may have been due to the higher sun exposure than women (25 ± 13 vs. 21 ± 11). It is important to highlight that 66% of the participants did not use sunscreen, which validates the results regarding sun exposure and 25OHD concentration. A study that included 95,137 Korean individuals analyzed 25OHD status according to sex, age, and season also reported differences in 25OHD status, with higher concentrations among the male participants [40].

The prevalence of inadequate micronutrient and macronutrient intakes was assessed previously in participants of the current study showing that 100, and 99% of the participants had inadequate vitamin D, and calcium intakes, respectively [41]. Therefore, evidently diet was not a significant external source of vitamin D that contributed to the variation in 25OHD concentration, according to the seasons.

Skin color and skin type did not influence 25OHD concentration, a finding discordant from those of previous studies [15, 42]. However, in our study, skin color was self-classified, which means that individuals subjectively classified themselves, leading to various potential biases; therefore, the reported skin color may not be the actual skin color.

The studied participants were sampled from a location with a steady and a high year-round solar radiation. This observation might explain, at least in part, the smaller magnitude of the coefficient of determination (10%) compared to previous investigations carried out in locations with larger variations in sunlight exposure among seasons.

Our study had some limitations, including its cross-sectional study design, in which the same individuals were not assessed over the four seasons and the number of the participants in each season. Moreover, the ultraviolet radiation and UVI measures reflected not only UVB radiation, but also UVA radiation. This correction may be performed in others studies considering assessments for vitamin D3 production in human skin from outdoor exposures as well as account related to different contributions of each action spectrum with changing solar zenith angle [43].

In addition, given the unmatched design, we cannot fully rule out that covariate imbalances might contribute to the observed associations. However, for the examined covariates, only two were considered statistically significant in univariable models, and associations between 25OHD and sunlight exposure score, seasons and body surface area were robust to adjustments for other covariates included in the model.

## Conclusions

In conclusion, sex, WHR, sun exposure and summer season were predictors of 25OHD status among individuals with MetS. In addition to this, HDL-cholesterol was the only component of the MetS that differed significantly among the seasons. These results demonstrate the importance of considering these variables in clinical interventions with vitamin D, and underscore the need for further development and updated guidelines for the treatment of MetS.

## Additional file

**Additional file 1.** Multiple regression model for the prediction of individuals with metabolic syndrome.

## Abbreviations

MetS: metabolic syndrome
25OHD: 25-Hydroxyvitamin D
UVI: ultraviolet radiation index
BMI: body mass index
WHR: waist:hip ratio
WC: waist circumference
PTH: parathyroid hormone
7-DHC: 7-Dehydrocholesterol
NCEP-ATP III: National Cholesterol Education Program-Adult Treatment Panel III
LAVAT: Laboratory of Tropical Environmental Variables
LDL: low-density lipoprotein
HDL: high-density lipoprotein
