# Supplementary material for: Predictors of 25-hydroxyvitamin D status among individuals with metabolic syndrome: a cross-sectional study
Source: Diabetol Metab Syndr. 2018 Jun 4;10:45. doi: 10.1186/s13098-018-0346-1 (PMC5987652; doi:10.1186/s13098-018-0346-1)
Supplement: Supplementary file 1 — Additional file 1. Multiple regression model for the prediction of individuals with metabolic syndrome. [file 13098_2018_346_MOESM1_ESM.docx]

Multiple regression model for the prediction of individuals with Mets 25OHD concentrations (ng/mL) (n = 180).

**Model 1:** variables related to sunlight exposure plus demographic variables and body surface area, captured as waist-to-hip ratio (p = 0.001)

| **25OHD (ng/mL)** | | | | | |
| --- | --- | --- | --- | --- | --- |
| **Predictors** | **β** | **SE** | ***95% CI*** | ***p*^a^** | ***R²*** |
| Sun exposure score | 0.164 | 0.06 | 0.05–0.28 | 0.006 | 0.104 |
| Seasons |  |  |  |  |  |
| Winter (Ref.) |  |  |  |  |  |
| Spring | 3.178 | 1.57 | 0.08–6.27 | 0.045 |  |
| Summer | 5.593 | 1.92 | 1.81–9.38 | 0.004 |  |
| Autumn | 3.108 | 2.29 | -1.42–7.64 | 0.18 |  |
| Gender | 3.716 | 1.67 | 0.43–7.00 | 0.027 |  |
| WHR | -25.080 | 11.23 | -47.26–2.89 | 0.027 |  |
| Age (years) | 0.0001 | 0.56 | -0.11–0.11 | 0.10 |  |
| Use of sunscreen |  |  |  |  |  |
| Do not use (Ref.) |  |  |  |  |  |
| Always use | 2.671 | 1.41 | -0.11–5.45 | 0.06 |  |
| Sometimes use | -3.91 | 8.91 | -21.52–13.69 | 0.66 |  |

β, regression coefficient; SE, standard error; CI, confidence interval; Ref., reference group. ^a^ p, for multiple comparisons: winter vs. spring, winter vs. summer, winter vs. autumn, spring vs. summer, spring vs. autumn, and summer vs. autumn.

**Model 2:** variables related to sunlight exposure plus demographic variables and body surface area, captured as body mass index (p = 0.005)

| **25OHD (ng/mL)** | | | | | |
| --- | --- | --- | --- | --- | --- |
| **Predictors** | **β** | **SE** | ***95% CI*** | ***P^a^*** | ***R²*** |
| Gender | 2.353 | 1.64 | -0.88 – 5.58 | 0.15 | 0.084 |
| Age | -0.050 | 0.60 | -0.17 – 0.07 | 0.41 |  |
| BMI (kg/m^2^) | -0.118 | 0.11 | -0.33 – 0.10 | 0.28 |  |
| Use of sunscreen |  |  |  |  |  |
| Do not use (Ref.) |  |  |  |  |  |
| Always use | 2.903 | 1.42 | 0.10 – 5.71 | 0.042 |  |
| Sometimes use | -2.064 | 8.90 | -19.65 – 15.53 | 0.82 |  |
| Sun exposure score | 0.135 | 0.58 | 0.02 – 0.25 | 0.021 |  |
| Seasons |  |  |  |  |  |
| Winter (Ref.) |  |  |  |  |  |
| Spring | 3.186 | 1.59 | 0.04 – 6.33 | 0.047 |  |
| Summer | 5.607 | 1.94 | 1.77 – 9.44 | 0.004 |  |
| Autumn | 3.553 | 2.32 | -1.03 – 8.14 | 0.13 |  |

β, regression coefficient; SE, standard error; CI, confidence interval; Ref., reference group. ^a^ p, for multiple comparisons: winter vs. spring, winter vs. summer, winter vs. autumn, spring vs. summer, spring vs. autumn, and summer vs. autumn.

**Model 3:** variables related to sunlight exposure plus demographic variables and body surface area, captured as waist circumference (p = 0.004)

| **25OHD (ng/mL)** | | | | | |
| --- | --- | --- | --- | --- | --- |
| **Predictors** | **β** | **SE** | ***95% CI*** | ***P^a^*** | ***R²*** |
| Gender | 2.706 | 1.61 | -0.47 – 5.88 | 0.09 | 0.088 |
| Age (years) | -0.041 | 0.06 | -0.15 – 0.07 | 0.47 |  |
| WC (cm) | -0.076 | 0.05 | -0.18 – 0.03 | 0.16 |  |
| Use of sunscreen |  |  |  |  |  |
| Do not use (Ref.) |  |  |  |  |  |
| Always use | 2.733 | 1.43 | -0.08 – 5.55 | 0.06 |  |
| Sometimes use | -2.517 | 8.91 | -20.12 – 15.09 | 0.78 |  |
| Sun exposure score | 0.140 | 0.058 | 0.03 – 0.25 | 0.017 |  |
| Seasons |  |  |  |  |  |
| Winter (Ref.) |  |  |  |  |  |
| Spring | 3.221 | 1.59 | 0.087 – 6.36 | 0.044 |  |
| Summer | 5.663 | 1.94 | 1.84 – 9.49 | 0.004 |  |
| Autumn | 3.466 | 2.31 | -1.09 – 8.02 | 0.14 |  |

β, regression coefficient; SE, standard error; CI, confidence interval; Ref., reference group. ^a^ p, for multiple comparisons: winter vs. spring, winter vs. summer, winter vs. autumn, spring vs. summer, spring vs. autumn, and summer vs. autumn.

**Model 4:** demographic variables **(**p = 0.49)

| **25OHD (ng/mL)** | | | | | |
| --- | --- | --- | --- | --- | --- |
| **Predictors** | **β** | **SE** | ***95% CI*** | ***P*** | ***R²*** |
| Gender | 2.954 | 1.68 | 0.36 – 6.27 | 0.08 | 0.025 |
| Age | -0.026 | 0.06 | -0.14 – 0.09 | 0.65 |  |
| Region |  |  |  |  |  |
| East (Ref.) |  |  |  |  |  |
| Agreste | 1.370 | 2.28 | -3.14 – 5.88 | 0.55 |  |
| Central | -2.864 | 3.03 | -8.85 – 3.13 | 0.35 |  |
| West | 2.243 | 5.41 | -8.43 – 12.91 | 0.68 |  |

β, regression coefficient; SE, standard error; CI, confidence interval; Ref., reference group.

**Model 5:** variables related to sunlight exposure only (p = 0.13)

| **25OHD (ng/mL)** | | | | | |
| --- | --- | --- | --- | --- | --- |
| **Predictors** | **β** | **SE** | ***95% CI*** | ***P*** | ***R²*** |
| Seasons |  |  |  |  | 0.000 |
| Winter (Ref.) |  |  |  |  |  |
| Spring | 3.118 | 1.10 | -0.82 – 7.06 | 0.12 |  |
| Summer | 5.413 | 2.06 | 1.35 – 9.47 | 0.009 |  |
| Autumn | 3.402 | 2.46 | -1.45 – 8.25 | 0.17 |  |
| UVI | 0.003 | 0.46 | -0.91 – 0.91 | 0.10 |  |
| Sun exposure score | 0.426 | 0.60 | 0.24 – 0.26 | 0.019 |  |
| Skin type |  |  |  |  |  |
| I (Ref.) |  |  |  |  |  |
| II | -1.847 | 2.83 | -7.44 – 3.75 | 0.52 |  |
| III | -3.943 | 3.01 | -9.88 – 1.99 | 0.19 |  |
| IV | -1.705 | 3.03 | -7.69 – 4.28 | 0.57 |  |
| V | -2.421 | 3.24 | -8.82 – 3.98 | 0.46 |  |
| VI | -8.880 | 9.50 | -27.64 – 9.88 | 0.35 |  |
| Self-referred skin color |  |  |  |  |  |
| Black |  |  |  |  |  |
| Mixed | 0.646 | 3.03 | -5.34 – 6.64 | 0.83 |  |
| White | -0.002 | 3.34 | -6.59 – 6.59 | 1.000 |  |
| Yellow | 1.397 | 4.70 | -7.88 – 10.68 | 0.77 |  |
| Indigenous | -2.052 | 9.59 | -20.98 – 16.88 | 0.83 |  |
| Use of sunscreen |  |  |  |  |  |
| Do not use (Ref.) |  |  |  |  |  |
| Always use | 2.656 | 1.49 | -0.29 – 5.60 | 0.08 |  |
| Sometimes use | -4.334 | 9.69 | -23.51 – 14.84 | 0.66 |  |

β, regression coefficient; SE, standard error; CI, confidence interval; Ref., reference group.

**Model 6:** total serum calcium and PTH (p = 0.11)

| **25OHD (ng/mL)** | | | | | |
| --- | --- | --- | --- | --- | --- |
| **Predictors** | **β** | **SE** | ***95% CI*** | ***P*** | ***R²*** |
|  |  |  |  |  | 0.000 |
| Total serum calcium (mg/dL) | -1.428 | 0.98 | -3.37 – 0.51 | 0.15 |  |
| PTH (pg/mL) | -0.043 | 0.31 | -0.10 – 0.02 | 0.16 |  |

β, regression coefficient; SE, standard error; CI, confidence interval; Ref., reference group.

**Model 7:** nutrition-related variables (p = 0.78)

| **25OHD (ng/mL)** | | | | | |
| --- | --- | --- | --- | --- | --- |
| **Predictors** | **β** | **SE** | ***95% CI*** | ***P*** | ***R²*** |
|  |  |  |  |  | 0.000 |
| Vitamin D intake | -0.383 | 0.26 | -0.54 – 0.47 | 0.88 |  |
| Calcium intake | 0.002 | 0.00 | -0.00 – 0.01 | 0.48 |  |

β, regression coefficient; SE, standard error; CI, confidence interval; Ref., reference group.

**Supplementary table -** Linear regression model for the prediction of individuals with MetS 25OHD concentrations (ng/mL) (n = 180).

| **25OHD (ng/mL)** | | | | | |
| --- | --- | --- | --- | --- | --- |
| **Predictors** | **β** | **SE** | ***95% CI*** | ***p*^a^** | ***R²*** |
| Gender | 2.721 | 1.65 | -0.53 – 5.97 | 0.10 | 0.010 |
| Total serum calcium (mg/dL) | -1.562 | 0.98 | -3.49 – 0.37 | 0.11 | 0.000 |
| PTH (pg/mL) | -0.047 | 0.03 | -0.11 – 0.02 | 0.14 | 0.000 |
| WHR | -9.391 | 10.43 | -29.97 – 11.19 | 0.37 | -0.001 |
| WC (cm) | -0.040 | 0.05 | -0.14 – 0.06 | 0.45 | -0.002 |
| Calcium intake | 0.002 | 0.003 | -0.003 – 0.006 | 0.49 | 0.003 |
| BMI (kg/m^2^) | -0.064 | 0.10 | -0.26 – 0.13 | 0.52 | -0.003 |
| Age (years) | -0.018 | 0.06 | -0.13 – 0.09 | 0.75 | -0.005 |
| Vitamin D intake | 0.028 | 0.24 | -0.44 – 0.50 | 0.91 | 0.000 |

β, regression coefficient; SE, standard error; CI, confidence interval; Ref., reference group.

**Supplementary table continuation -** Linear regression model for the prediction of individuals with MetS 25OHD concentrations (ng/mL) (n = 180).

| **25OHD (ng/mL)** | | | | | |
| --- | --- | --- | --- | --- | --- |
| **Predictors** | **β** | **SE** | ***95% CI*** | ***p*^a^** | ***R²*** |
| Seasons |  |  |  |  | 0.042 |
| Winter (Ref.) |  |  |  |  |  |
| Spring | 2.994 | 1.59 | -0.16 – 6.15 | 0.063 |  |
| Summer | 5.924 | 1.95 | 2.07 – 9.78 | 0.003 |  |
| Autumn | 3.882 | 2.33 | -0.71 – 8.47 | 0.10 |  |
| IRUV | 0.592 | 0.37 | -0.14 – 1.32 | 0.11 | 0.001 |
| Region |  |  |  |  | 0.007 |
| East (Ref.) |  |  |  |  |  |
| Agreste | 0.969 | 2.24 | -3.46 – 5.39 | 0.67 |  |
| Central | -2.773 | 3.01 | -8.70 – 3.16 | 0.36 |  |
| West | 1.848 | 5.36 | -8.74 – 12.43 | 0.73 |  |
| Sun exposure score | 0.155 | 0.06 | 0.04 – 0.27 | 0,008 | 0.034 |
| Skin type |  |  |  |  | -0.014 |
| I (Ref.) |  |  |  |  |  |
| II | -1.471 | 2.75 | -6.90 – 3.95 | 0.59 |  |
| III | -3.043 | 2.84 | -8.66 – 2.57 | 0.29 |  |
| IV | -0.925 | 2.86 | -6.57 – 4.72 | 0.74 |  |
| V | -0.876 | 3.05 | -6.90 – 5.15 | 0.78 |  |
| VI | -9.592 | 9.54 | -28.42 – 9.23 | 0.32 |  |
| Self-referred skin color |  |  |  |  | -0.019 |
| Black |  |  |  |  |  |
| Mixed | 1.601 | 3.05 | -4.42 – 7.63 | 0.60 |  |
| White | 1.759 | 3.19 | -4.54 – 8.06 | 0.58 |  |
| Yellow | 2.751 | 4.55 | -6.24 – 11.74 | 0.55 |  |
| Indigenous | -2.150 | 9.69 | -21.28 – 16.98 | 0.83 |  |
| Use of sunscreen |  |  |  |  | 0.000 |
| Do not use (Ref.) |  |  |  |  |  |
| Always use | 2.408 | 1.46 | -0.48 – 5.29 | 0.10 |  |
| Sometimes use | -3.695 | 9.94 | -23.37 – 15.99 | 0.71 |  |

β, regression coefficient; SE, standard error; CI, confidence interval; Ref., reference group.
